# Editorial: Recent advances in the treatment of upper urinary tract bladder cancers

**DOI:** 10.3389/fruro.2023.1300741

**Published:** 2023-10-12

**Authors:** Łukasz Zapała, Aleksander Ślusarczyk

**Affiliations:** Clinic of General, Oncological and Functional Urology, Medical University of Warsaw, Warsaw, Poland

**Keywords:** bladder cancer, NMIBC (non-muscle invasive bladder cancer), MIBC (muscle invasive bladder cancer), immunotherapy, adjuvant

The results of the treatment of urothelial cancers affecting upper urinary tracts and, mainly, bladder, present the view of suboptimal management of the disease, with approximately 50% of patients undergoing radical cystectomy for muscle-invasive bladder cancer not surviving 5 years (1). Even non-muscle invasive bladder tumors are associated with suboptimal outcomes, including a significant risk of long-term cancer-specific mortality, which is particularly pronounced in patients with T1HG disease (2). Thus, the unmet need of modern urooncology is the sufficient adjuvant treatment in the perioperative setting. Recent advancements in the immunotherapy allow us to draw preliminary conclusions on adjuvant treatment to be implemented in new clinical scenarios, including BCG-unresponsive non-muscle invasive disease of the bladder. The results of randomized controlled trials (RCTs) involving immune checkpoint inhibitors (ICI) in the adjuvant setting after radical cystectomy for muscle-invasive bladder cancer (MIBC) are conflicting and though further research is needed for conclusive evidence (3). Recent progress in the minimally invasive surgical techniques have prompted the revision of concepts on the treatment of tumors located within upper urinary tract, as well.

This Research Topic focused on novel approaches in urothelial cancers, including upper urinary tract and bladder tumors through one original research (Bracarda et al.), two systematic reviews and meta-analysis (Liu et al., and Zeng et al.) and one case report (Mao et al.).

Bracarda et al. focused on a unique project aiming at redesigning the current model of care for advanced urothelial carcinoma patients to identify limitations and recommend future actions. In the group of panel experts, the analysis of the two scenarios as part of a multidimensional consensus process was performed, reaching for the recommendations for specific domains of the disease, while Delphi methodology was used to establish consensus among the panelists. This is the representation of the search for the rethinking of the current management and treatment model for advanced urothelial carcinoma, which nowadays often leads many patients to abandon treatment as a consequence of limited treatment options with poor tolerability. On the other hand, a multidisciplinary approach would be often the answer for an early and effective diagnosis and improved quality of life of the oncologic patients. While novel treatment modalities impose great value for the patients’ life, clinicians are forced to balance this positive clinical impact with the economic implications of the new treatment options. The paper represents an innovative analysis model for other healthcare systems or countries. As a result, U-CHANGE Project was proved to be easily applicable to local level, indicating a minimum level of acceptance: accuracy, adequate medical education for patients and caregivers, and access to innovative therapeutical tools for a more efficient patient care.

In the paper by Mao et al. a recent bladder-preserving strategy was presented and encompassed multimodal treatment including maximal transurethral resection of the tumor (TURBT) combined with chemotherapy plus immune checkpoint inhibitor - tislelizumab. The current papers on bladder-preserving techniques introduce the new concept of an alternative to radical cystectomy, which is a truly debilitating procedure with a significant impact on patients’ quality of life in one hand, and with often unsatisfactory oncological results for advanced MIBC at the same time (4). However, the present trimodal bladder-preserving treatment has well-known drawbacks, e.g. some patients experience reduced bladder capacity and develop overactive bladder (5). Tislelizumab is a novel humanized monoclonal antibody programmed death receptor-1 (PD-1) inhibitor, which proved its applicability in the single-arm phase 2 trial (NCT04004221/CTR2017007) in the treatment of patients with metastatic urothelial cancer and high PD-L1 expression, who had failed platinum-based chemotherapy regimens (6). The authors reported two cases diagnosed with recurrent MIBC who achieved pathological complete response and bladder-preserving after maximal TURBT combined with chemotherapy plus tislelizumab. In the above cases, the authors replaced the postoperative concurrent chemoradiotherapy with tislelizumab until the end of follow-up without MIBC recurrence in both patients, suggesting that tislelizumab may be efficacious in the multimodal management for selected MIBC patients, although randomized trials are required to confirm that findings.

Liu et al. performed a meta-analysis that is an update of the previous one published by Hu et al. (7), aiming mainly on the effect of long-term use of metformin on oncological results of the bladder cancer treatment. There were 12 retrospective cohort studies on the association between the usage of metformin and the incidence or oncologic outcomes of bladder cancer included. The authors concluded that metformin could decrease the incidence and prolong recurrence-free survival of bladder cancer but showed no significant protective effects for overall survival and progression-free-survival. Thus, metformin may emerge as preferred antidiabetic drug for high-risk bladder cancer patients with type 2 diabetes mellitus, which could simultaneously decrease the recurrence risk.

Current postoperative adjuvant management of non-muscle invasive bladder cancer comprises intravesical bacillus Calmette–Guéarin (BCG) immunotherapy and intravesical chemotherapy (8). Due to the limitations of BCG therapy, novel drugs and strategies are awaited (9). Zeng et al. reported the results of the network meta-analysis, which showed that hyperthermia intravesical chemotherapy (HIVEC) performed slightly better than BCG in preventing recurrence but demonstrated lower efficacy in preventing tumor progression, although both differences were not statistically significant. However, in the subgroup analysis of studies involving highly predominant male population and ≥ 2.5 year of follow-up, a significantly better RFS for HIVEC compared to BCG was observed. This suggests that HIVEC offers an alternative to BCG for reducing tumor recurrence rates in male patients.

In summary, there is a growing need to improve the current landscape of treatment modalities of urothelial cancers, both in the aspect of management guidelines and novel therapies. As elucidated in this editorial, we have provided a concise introduction to a series of papers that collectively illuminate the evolving frontier of urothelial cancer care. The challenges posed by this disease necessitate a multi-faceted approach, encompassing not only the optimization of established protocols but also the investigation and integration of novel therapeutic modalities. In the face of these challenges, ongoing studies will undoubtedly lead to improved outcomes, enhanced quality of life, and renewed hope for those affected by urothelial cancer.

## Author contributions

ŁZ: Writing – original draft, Writing – review & editing. AŚ: Writing – original draft, Writing – review & editing.
